# Rural–urban disparities in child nutrition in Tabora, Tanzania: a socioeconomic decomposition and implications for food security policy

**DOI:** 10.3389/fnut.2026.1800873

**Published:** 2026-07-20

**Authors:** Felician Andrew Kitole, Temitope Oluwaseun Ojo, Olutosin Ademola Otekunrin, Theresa Abosede Ojo

**Affiliations:** 1 Department of Economics, Mzumbe University, Morogoro, Tanzania; 2 Department of Business and Social Sciences, Faculty of Agriculture, Dalhousie University, Halifax, NS, Canada; 3 Department of Plant, Food and Environmental Sciences, Faculty of Agriculture, Dalhousie University, Halifax, NS, Canada; 4 Disaster Management Training and Education Centre for Africa, University of the Free State, Bloemfontein, South Africa; 5 Department of Agribusiness, Federal University of Agriculture and Development Studies, Iragbiji (FUADSI), Nigeria; 6 Innovation Lab for Policy Leadership in Agriculture and Food Security (PiLAF), University of Ibadan, Ibadan, Nigeria

**Keywords:** Blinder-Oaxaca analysis, child health, child nutrition, socioeconomic disparities, Tanzania

## Abstract

Child malnutrition remains a major barrier to human capital development in sub-Saharan Africa, yet the drivers of rural–urban inequalities remain insufficiently understood. This study examines socioeconomic disparities in child health between rural and urban households in Tabora, Tanzania, using cross-sectional data from 490 households across seven districts. Employing Blinder–Oaxaca decomposition, instrumental variable (IV) estimation, and structural equation modeling (SEM), the study finds a pronounced rural–urban gap in child nutrition, with a Height-for-Age Z-score difference of −0.685, indicating significantly higher stunting prevalence in rural areas. The decomposition results show that approximately 72% of the observed gap is explained by differences in observable characteristics, with household income, nutrition intake, and drinking water practices (boiling water consumption) emerging as the most influential contributors, highlighting the central role of economic constraints and basic infrastructure deficits rather than purely behavioral differences. SEM and IV results further confirm that these factors operate through interconnected pathways affecting both objective nutrition outcomes and perceived health status, while parental education plays a comparatively limited role. Based on these findings, policy interventions should prioritize income-enhancing strategies such as rural livelihood support, targeted cash transfers, and employment programs, alongside large-scale investment in rural water infrastructure, including piped water systems, boreholes, and household water treatment solutions, given their substantial contribution to the health gap. In addition, nutrition-specific interventions such as community-based dietary education, school feeding programs, and distribution of fortified foods should be strengthened to directly address inadequate nutrition intake. These efforts should be complemented by expanded rural healthcare outreach and integrated intersectoral coordination between health, water, and economic development sectors to ensure that improvements in income, nutrition, and water access are simultaneously addressed, thereby producing sustained reductions in child health inequalities.

## 1 Introduction

Preventable child mortality remains unacceptably high, with recent statistics indicating “an estimated 4.9 million children died before their fifth birthday in 2024, including 2.3 million newborns” (1). Most occur in Sub-Saharan Africa and Southern Asia, reflecting entrenched inequities in access to essential health and nutrition services (1, 2). Since the nineteenth century, from Engels to Salvador, child health has been understood as socially patterned rather than biologically random (3). Despite definitional differences, health inequality, disparity, and discrepancy consistently denote systematic variation by socioeconomic position (4, 5). These gradients continue to structure survival in early life. Their persistence reflects weak redistribution mechanisms in health and social systems. Understanding them requires tracing how social stratification is converted into biological vulnerability.

In Tanzania, child health reflects persistent spatial and socioeconomic stratification (6). National survey data show marked regional variation in diarrhea and anemia, signalling unequal access to prevention, early diagnosis, and treatment (1, 7). Although public health investment has expanded, its translation into population-level equity remains limited (8, 9). Health financing has not corrected structural service imbalances. Rural populations continue to face constrained infrastructure, delayed care, and reduced service availability. Urban areas benefit from comparatively denser health and nutrition systems. These divergences produce stable rural–urban health gradients in children.

Socioeconomic status (SES), defined by income, education, and occupation, determines exposure to material and institutional advantage (10). It structures access to nutrition, healthcare, and safe environments during critical developmental periods. Resource deprivation constrains care-seeking and dietary adequacy (11). Parental disadvantage further limits human capital investment in children. These exposures accumulate, producing durable inequality in growth trajectories. SES therefore operates as a primary upstream determinant of child health gradients (12, 13).

Adverse birth outcomes, including low birth weight and prematurity, are concentrated in socioeconomically deprived households (14). Empirical assessment of child health commonly relies on parental education, employment, and income as proxies for structural position (15). These indicators capture upstream determinants rather than proximate clinical conditions (16). Disadvantage across these domains generates persistent deficits across the life course (17). Constraints in nutrition, healthcare access, and environmental safety interact cumulatively (18). The result is stratified health trajectories from birth through adolescence. Socioeconomic conditions thus account for a substantial proportion of observed heterogeneity in child health (19).

In Tanzania, evidence remains dominated by anthropometric indicators that capture growth but not perceived or functional health. This study addresses this limitation by integrating height-for-age (HAZ) with self-reported health (SRH), combining objective and subjective health dimensions. This dual approach improves identification of socioeconomic gradients in child health. The analysis focuses on rural–urban disparities in Tabora, a region characterised by pronounced differences in service access and living conditions. Prior Tanzanian studies have examined parenting and spatial variation in child health (20, 21), yet remain methodologically narrow in health measurement. Evidence from Ghana (22, 23) and China (24, 25) similarly privileges nutritional outcomes, under-representing multidimensional health constructs. By integrating HAZ and SRH, this study captures both biological status and perceived health functioning. The findings strengthen evidence on how socioeconomic stratification translates into unequal child health and may inform equity-oriented policy design in Tanzania.

## 2 Theoretical foundation

The theoretical framework underpinning this study draws heavily from Human Capital Model for Health as developed by Grossman (26). The model views health as a form of human capital in which individuals invest to improve productivity and wellbeing. Within this framework, good health is a valuable asset that increases the time and capacity individuals can devote to both market and non-market activities.

In accordance with Grossman’s conceptualization, individuals make decisions to invest in health capital until the cost of acquiring an additional unit of health capital equals the combination of the additional time spent in production and the cost incurred when healthy. Features that prove beneficial to individuals in this context encompass:


$$U=U(H_{t},Z_{t})$$

Within this framework $H_{t}$ signifies the stock of health in period t , and $Z_{t}$ denotes the consumption of other goods, the perspective assumes a fixed and endogenous length of life. Despite the unavoidable decline in an individual’s stock of life throughout their lifespan, the individual retains the capacity to make investments in health. This is encapsulated by the health investment equation:


$$\frac{\delta H_{t}}{\partial t}=I_{t}-\delta_{t}H_{t}$$

Here, $I_{t}\;$ *t* is the gross investment in health, and $\delta_{t}$ is the rate of depreciation of health during period t . The gross investment in health ( $I_{t}$ ) is produced by an individual, while $Z_{t}$ represents other commodities produced. Thus, the production function for health investment ( $I_{t}$ ) and other commodities $Z_{t}$ are given by:


$$I_{t}=I_{t}\;(M_{t};h_{t,H};E_{t})$$

$$Z_{t}=Z_{t}(X_{t};h_{t,Z};E_{t})$$

Whereas $M_{t}$ represents healthcare, $X_{t}$ denotes market goods, $h_{t,H}$ and $h_{t,Z}$ are time inputs, and $E_{t}$ is the human capital stock. Changes in human capital contribute to the production efficiency of non-market goods within an economy.

While the Human Capital Model of Health developed by Michael Grossman is useful in explaining health as an investment that improves productivity, it has some limitations. The model mainly focuses on individual choices and household investments in health, while giving less attention to broader structural factors such as poverty, inequality, sanitation, food access, and place of residence. It also assumes that households have equal ability to make health investments, which is often unrealistic in low-income rural settings.

To address these limitations, this study is also guided by the Social Determinants of Health (SDH). This framework explains that health and nutrition outcomes are shaped by the social, economic, and environmental conditions in which people live. Factors such as household income, parental education, access to healthcare (clinic visists), and rural - urban residence influence child nutrition status. In this study, the framework helps explain how differences in living conditions between rural and urban households in Tabora contribute to disparities in child nutrition outcomes.

**Figure 1:**
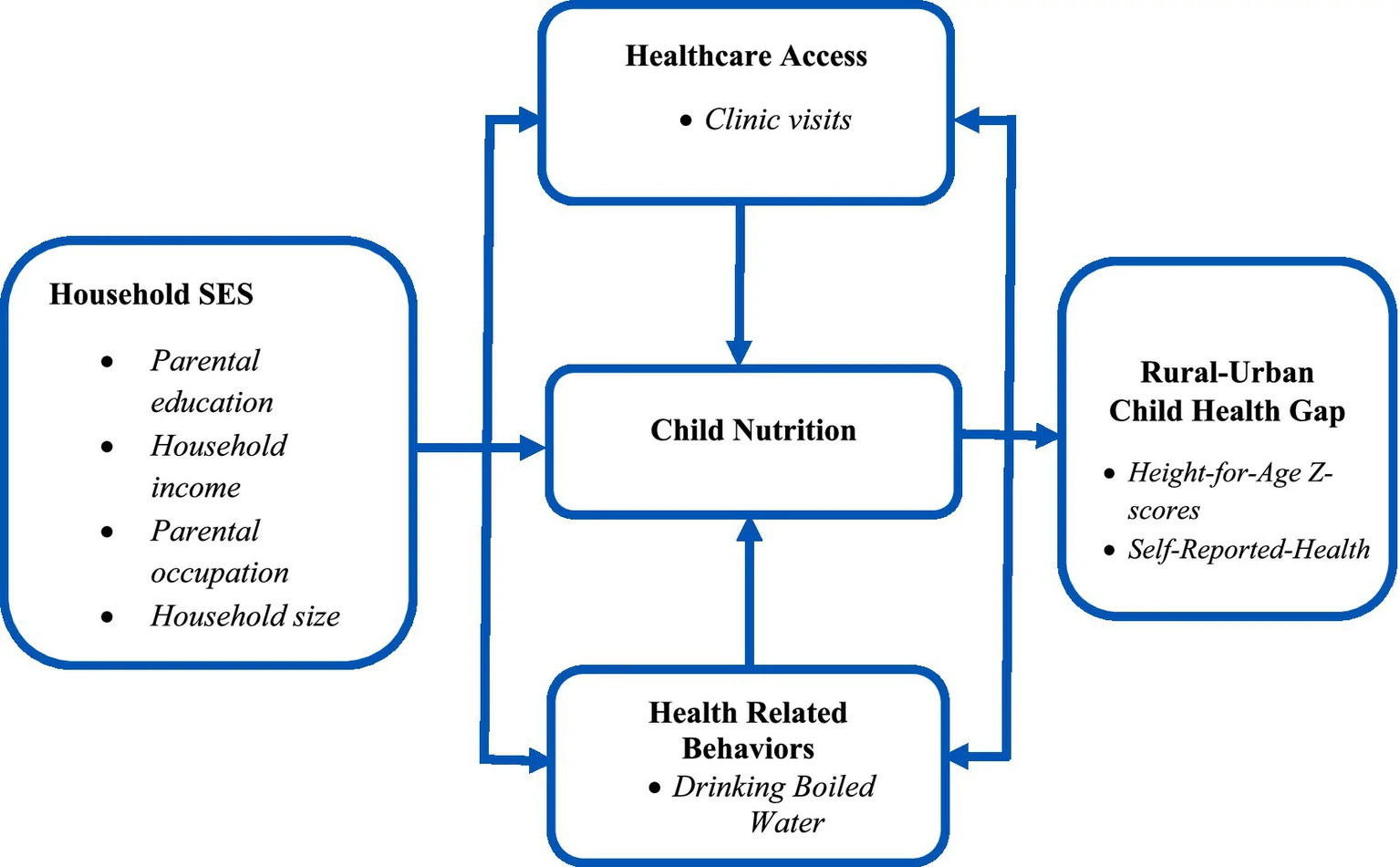
Conceptual framework of the study.

Figure 1 presents the conceptual framework of this study, adapted from Liu et al. (16, 27), and grounded in both the Human Capital Model of Health developed by Michael Grossman and the Social Determinants of Health. The framework illustrates the causal relationships between household socioeconomic status (SES), mediating factors, and rural–urban disparities in child health outcomes. Household SES variables, including parental education, household income, parental occupation, and household size, are treated as the main underlying determinants of child health. In line with Grossman’s model, health is viewed as a productive asset shaped through household investments in nutrition, healthcare, and behaviours that improve wellbeing. Accordingly, the effects of SES operate through key intermediate factors, namely healthcare access, child nutrition, and health-related behaviours, which reflect differences in households’ ability to obtain health services, provide adequate nutrition, and adopt practices that promote child wellbeing. At the same time, the Social Determinants of Health framework justifies the inclusion of broader contextual factors such as poverty and rural–urban residence, which influence access to resources and opportunities. These pathways ultimately affect child health outcomes measured by indicators such as Height-for-Age Z-scores and self-reported health status. Thus, the framework suggests that rural–urban child health gaps arise both directly from socioeconomic inequalities and indirectly through unequal access to health-promoting conditions (28).

## 3 Research methodology

### 3.1 Study area

Tabora Region is located in central Tanzania between 4° and 7° south of the Equator and lies on the central plateau at an elevation of approximately 1,000–1,500 meters above sea level. It is the largest region in the country, covering 76,278 km^2^. According to the 2022 national census, the region has a population of 3,391,679, up from 2,291,263 in 2012. Administratively, Tabora comprises seven districts: Igunga, Kaliua, Nzega, Sikonge, Tabora Urban, Urambo, and Uyui.

Child health indicators in the region remain below national standards. The 2015/2016 Tanzania Demographic and Health Survey (TDHS) reported low vaccination coverage of 48.97%, compared with the national average. Recent estimates indicate a child malnutrition prevalence of 51%, exceeding both the national average (30–32%, stunted growth) and the global average (23.2%) (29, 30). These conditions highlight persistent child health challenges across the region. Accordingly, this study includes all seven districts to ensure comprehensive regional coverage and improve the robustness of the analysis.

### 3.2 Research design and sample size estimation

This study used a cross-sectional approach to examine the impact of socioeconomic status on child health inequalities, comparing rural and urban areas in the Tabora region. This design, which includes all seven states in the region, was chosen for its ability to capture multiple outcomes simultaneously. In addition, the cross-sectional methodology facilitates the collection of self-reported data on child health (SCH), an important aspect that requires careful measurement but is often neglected in existing child health datasets.

The study focused on households with children under 5 years of age, with a particular emphasis on children aged 2 to 5 years. This age category was chosen because it corresponds to a critical stage in physical development, when children move from anxiety to active exploration of the environment. To qualify, families must live in the same household, share food cooked in the same pot and have at least one child under the age of five. According to the 2022 Tanzania census, a total of 680,103 households live in Tabora district. This study assumed that at least one person in each household met the inclusion criteria. This assumption is to ensure a sufficient number of households to provide the necessary information for research purposes while minimizing household selection bias. For larger population sizes, the sample size formula proposed by Yamane (31).


$$n=\frac{N}{1+N\;(e^{2})}=\frac{680,103}{1+680,103\;(0.05^{2})}=399.76488\approx 400$$

The sample size was adjusted to include 490 participants, with 70 households randomly selected from each of the seven districts of the Tabora region to ensure fair representation. Table 1 describes the study variables and their measures and provides a clear and transparent view of the study structure. It serves as a useful reference for understanding the research methodology and the importance of each variable on child health disparities between rural and urban areas in the Tabora region.

**Table 1:** Variable measurement and description.

Name of the variable	Variable definition	Measurement	Expected relationship
Height-for-age Z-score	This is the number of standard deviations from the median height of a reference population.	Z-score	NA
Self-reported health status (SRH)	Parent-reported health status for children aged 2–5 years (i.e., 1 = Good health, 0 Otherwise)	Dummy	NA
Level of education	Dummy variables are used,1 for uneducated, 2 for primary education, 3 for secondary education, 4 for college/university	Categorical	+/−
Parental Occupation group	Dummy if a parent is a farmer. 1 if a parent is farmer, o for non-farmer occupation	Dummy	+/−
Household size	Number of people residing in a given family.	Continuous	−
Household income monthly	Average monthly income earned by parents of children under 5 years old, measured by their total monthly earnings.	Tshs	+
Access to healthcare	1 if a child visited clinic regularly, 2 for rarely clinic visit, 3 if a child does not visit clinic	Categorical	+
Nutrition intake	1 if a child was fed the nutritious food in the last 2 weeks, 2 if rarely fed nutritious food, 3 if not fed nutritious food regularly	Categorical	+
Boiled drinking water	1 if parents boil drinking water, 0 otherwise	Dummy	

### 3.3 Data analysis

This study examines disparities in child health between rural and urban children in the Tabora region by applying a range of complementary econometric techniques to capture the complex relationships between socioeconomic conditions and health outcomes. Descriptive statistics are first used to summarize the main characteristics of the sample and highlight differences across place of residence. Ordinary Least Squares (OLS) regression models are then employed to estimate the effects of socioeconomic status on children’s nutritional wellbeing, measured by Height-for-Age Z-scores (HAZ), while Probit models are used to analyse the determinants of children’s Self-Reported Health (SRH), with results interpreted through marginal effects.

To address the multidimensional nature of child health, the study further applies Structural Equation Modelling (SEM), which enables the simultaneous estimation of direct and indirect relationships among socioeconomic status, mediating factors, and health outcomes. In this framework, household socioeconomic characteristics are specified as the main exogenous variables, while healthcare access, child nutrition, and health-related behaviours are treated as intermediary channels through which socioeconomic inequalities affect HAZ and SRH. SEM is particularly appropriate because it combines features of multiple regression, confirmatory factor analysis, and path analysis, thereby allowing both observed and latent constructs to be examined within one coherent model. Mediation analysis is also conducted to quantify the indirect effects of socioeconomic determinants on child health outcomes.

Given the possibility of endogeneity especially in healthcare utilization, where households with unhealthy children are more likely to seek treatment, the study employs an instrumental variable approach mainly the Two Stage Least Squares (2SLS). The instrument used is the distance to the nearest health facility, which is strongly correlated with healthcare access because households located closer to clinics or dispensaries face lower travel costs, shorter travel time, and fewer barriers to obtaining care. At the same time, conditional on other household and community controls, distance is assumed not to affect child health outcomes directly except through its influence on access to healthcare services. This helps isolate the causal effect of healthcare access on child nutrition and health outcomes. The instrument qualifies the exclusion restriction (exogeneity), relevance, exclusivity (no direct effect), and monotonicity (see Appendix Tables A4, A5, A6)

Finally, rural–urban differences in child health are decomposed using the Oaxaca–Blinder framework (32) and the Fairlie decomposition technique (33). These methods separate observed disparities into two components: (i) the endowment effect, arising from differences in characteristics such as income, education, and household conditions, and (ii) the structural effect, reflecting differences in how these characteristics translate into health outcomes across rural and urban settings.

#### 3.3.1 Anthropometric measurement

The nutritional status of children in this study was gauged using Height-for-age-Z scores, denoted as Z-score. This score is computed as $Z\;\text{score}=\frac{T_{i}-T_{\text{median}}}{\sigma_{T}}$ whereas $\;T_{i}$ is the height of child; $T_{\text{median}}\;$ is the median height of the reference population of the same age and sex, and $\sigma_{T}$ is the standard deviation from the median of the reference population. This variable serves as one of the dependent variables in the child health equation.

The assessment of stunting, defined as short height for age, adhered to a scale established by the WHO (34). If the height-for-age Z-score falls below 2 standard deviations from the reference population mean, the child is categorized as stunted or chronically permanently disabled. In this study, this condition was coded as 1, otherwise as 0. Both Z-scores and height-for-age (HAZ) contribute to the evaluation of child health, serving a dual purpose. This dual assessment facilitates the comparison of the two metrics, ensuring that the results are not unduly influenced by the specific model utilized for each indicator. This methodological choice was deliberate, aiming to enhance the reliability and validity of the study results when appraising the health status of children in the Tabora region.

#### 3.3.2 Analytical modelling

Children’s nutritional status was measured using continuous height-for-age Z-scores. Accordingly, the empirical analysis applied the ordinary least squares (OLS) estimation technique. Building on Grossman’s health production framework, households are assumed to derive utility from child health (H), consumption goods that affect health outcomes (Y), and consumption goods that are neutral to health production (X) (35). Child well-being is determined by household consumption choices and the allocation of resources to health-related inputs, including healthcare services and other expenditures that directly or indirectly influence health production. Within this framework, the household utility function is specified as follows:


U=U(X,Y,H)

The influence of Y and Z on child health can be further illustrated as:


$$H=F(Y,Z,\mu ),F_{y},F_{z},F_{\mu }\neq 0$$

Where μ represents an unknown factor, akin to health endowment resulting from genetic or environmental effects. Consequently, the maximization of household utility is always subject to its budget constraint, as defined by:


$$I=XP_{x}+YP_{y}+ZP_{z}$$

Where $P_{x}$ , $P_{y}$ , and $P_{z}$ denote the prices of health-neutral and health-related consumption goods, and child health investment goods, respectively, while I represents income.

The frameworks outlined above underscore that parents cannot directly purchase child health. Rather, they must obtain other goods that serve as mechanisms or pathways to influence health. Moreover, aligning with the Grossman model, attaining optimal child health may require sacrificing the production of certain goods. In this study, the production of child health has been guided by the following function:


$$H_{C}=H_{C}(N;A;B_{H,};\mu )$$

In this pediatric HAZ study, “H” represents quantitative health outcomes and “N” represents health data and behaviors. The study considers access to health care, use of boiling water and food consumption as specific inputs. These inputs and actions are assumed to be under the control of the individual (parent). “A” includes socio-demographic characteristics such as the age and gender of the child. “BH” includes factors related to parents’ socioeconomic status, μ refers to characteristics that cannot be observed by the researcher, but some of which can be observed in the subjects. In addition, to understand differences in child health between rural and urban areas, we used Oaxaca and Blinder analyzes to estimate observed disparities in child health, accounting for disparities between the two population groups. The study specifically aims to compare rural and urban populations within the study groups as presented in different studies (35, 36), thus the child health equation below captures rural–urban differences in child outcomes.


$$CH_{\mathrm{ir}}=\beta_{\mathrm{ir}}X_{\mathrm{ir}}+\varepsilon_{i}$$

Where i represents an individual child, r signifies the child’s residential type, $CH_{\mathrm{ir}}\;$ denotes child health for urban/rural (HAZ for this study), $X_{i}$ encompasses the explanatory variable of child health, $\beta_{i}\;$ is the coefficient, and $\varepsilon_{i}$ is an error term. The Oaxaca-Blinder decomposition approach dissects the disparity in health outcomes between urban and rural children into the observed and unobserved portions, as depicted below:


$$\begin{array}{l} CH^{\text{urban}}-CH^{\text{rural}}=(\;{\bar{X}}^{\text{urban}}-{\bar{X}}^{\text{rural}})\\ {\hat{\beta }}^{\text{urban}}+{\bar{X}}^{\text{rural}}(\;{\hat{\beta }}^{\text{urban}}-{\hat{\beta }}^{\text{rural}}) \end{array}$$

$$\begin{array}{l} CH^{\text{rural}}-CH^{\text{urban}}=(\;{\bar{X}}^{\text{urban}}-{\bar{X}}^{\text{rural}})\\ {\hat{\beta }}^{\text{rural}}+{\bar{X}}^{\text{urban}}(\;{\hat{\beta }}^{\text{urban}}-{\hat{\beta }}^{\text{rural}}) \end{array}$$

$$CH^{\text{urban}}-CH^{\text{rural}}=\Delta \bar{X}{\hat{\beta }}^{\text{rural}}+\Delta \beta {\bar{X}}^{\text{urban}}$$

Whereby $\Delta \bar{X}={\bar{X}}^{\text{urban}}-{\bar{X}}^{\text{rural}}$ and $\Delta \hat{\beta }={\hat{\beta }}^{\text{urban}}-{\hat{\beta }}^{\text{rural}}.$ The right-hand portion (RHS) The coefficients remain unchanged and represent the portion of the gap that is explained by differences in observed characteristics between households. The second part is the unexplained part, which refers to unobserved behavioral effects even when observed characteristics are held constant. Mussa (37) distinguishes between explained and unexplained components of urban–rural health gaps, assuming comparable income levels as controlled characteristics. However, households with similar income may still differ in expenditure patterns due to unobserved behavioral and preference heterogeneity. For the nonlinear decomposition of self-reported health (SRH) in urban–rural comparisons, Fairlie’s (38) method was applied to account for binary outcome structures. SRH was coded as a binary variable, where 1 represents good/excellent health and 0 represents fair/poor health. The nonlinear model is expressed as $Y=F(X\hat{\beta })$ ; and the decomposition for group differences can be written as:


$$\begin{array}{l} {\bar{Y}}^{U}-{\bar{Y}}^{R}=[\sum_{i=1}^{N^{U}}\frac{F({X_{i}}^{U}{\hat{\beta }}^{R})}{N^{U}}-\sum_{i=1}^{N^{R}}\frac{F({X_{i}}^{R}{\hat{\beta }}^{R})}{N^{R}}]\\ +[\sum_{i=1}^{N^{U}}\frac{F({X_{i}}^{U}{\hat{\beta }}^{U})}{N^{U}}-\sum_{i=1}^{N^{U}}\frac{F({X_{i}}^{U}{\hat{\beta }}^{R})}{N^{U}}] \end{array}$$

The first component represents the explained portion of the gap, attributable to differences in observed characteristics between urban and rural households. The second component captures the unexplained portion, reflecting differences in coefficients associated with unobserved factors and behavioral responses. This approach allows separation of structural differences from behavioral heterogeneity in SRH outcomes.


$$\frac{1}{N^{R}}\sum_{i=1}^{N^{R}}F({\hat{\propto }}^{*}+{X_{1i}}^{U}{\hat{\beta }}_{1}^{*}+{X_{2i}}^{U}{\hat{\beta }}_{2}^{*})+F({\hat{\propto }}^{*}+{X_{1i}}^{R}{\hat{\beta }}_{1}^{*}+{X_{2i}}^{U}{\hat{\beta }}_{2}^{*})$$

Likewise, the contribution of $\;X_{2}$ can be expressed in form of;


$$\frac{1}{N^{R}}\sum_{i=1}^{N^{R}}F({\hat{\propto }}^{*}+{X_{1i}}^{U}{\hat{\beta }}_{1}^{*}+{X_{2i}}^{U}{\hat{\beta }}_{2}^{*})+F({\hat{\propto }}^{*}+{X_{1i}}^{R}{\hat{\beta }}_{1}^{*}+{X_{2i}}^{R}{\hat{\beta }}_{2}^{*})$$

The role of each variable in the gap is essentially the change in the estimated mean probability that would occur if the urban residential distribution of that variable were replaced by a rural residential distribution and the distributions of the other variables were left unchanged.

## 4 Results

### 4.1 Description of respondents characteristics

The summary statistics results on Table 2 suggest substantial socioeconomic and nutritional variation among households in Tabora. Average household income is about 751,000, but the very wide range from as low as 2,000 to as high as 1,500,000 indicates stark income inequality, which likely translates into uneven access to food and health services. Household size averages 7 members, with some households reaching up to 12, implying a high dependency burden that can strain available resources. On average, each household has about 2 children, though some have as many as 5, further intensifying competition for food within families. The mean Height-for-Age Z-score of −1.96 is particularly concerning, as it is close to the threshold for stunting, signaling widespread chronic under nutrition. The minimum Z-score of −6.11 reflects cases of severe growth faltering among some children. Average child height is 91.4 cm, but the variation suggests disparities in physical development consistent with nutritional inequalities. Children in the sample are between 24 and 58 months old, with an average age of 41 months, a critical period for growth and development.

**Table 2:** Summary statistics.

Variable	Mean	Minimum	Maximum
Household Income	751,000	2000	1,500,000
Household size	7	2	12
Child number	2	1	5
Height-for-Age Zscore	−1.96	−6.11	2.19
Child height	91.4	71.8	111
Age of a child (months)	41	24	58

Additionally, the respondent characteristics in Table 3 reveal important socioeconomic and educational patterns that shape child nutrition outcomes in Tabora. A large majority of mothers (61.4%) and fathers (63.0%) have only primary education, while very few have attained college or university education (2.6% for mothers and 5.5% for fathers), indicating generally low human capital levels. Notably, 12% of mothers and 7.8% of fathers are uneducated, which may limit knowledge of proper childcare, feeding practices, and health-seeking behavior. In terms of livelihoods, 43.5% of parents are engaged in farming, while a slightly higher proportion (56.5%) are involved in non-farm activities, suggesting some diversification of income sources but still a strong reliance on agriculture. This mix of low education and semi-subsistence economic activity may constrain households’ ability to ensure consistent and nutritious diets for children, especially in rural settings where access to services and markets is limited.

**Table 3:** Respondents characteristics.

Variable	Observation	Mean	Percentage
Mother education			
Uneducated	490	0.12	12.0
Primary education		0.61	61.4
Secondary education		0.24	24.0
College/University education		0.03	2.6
Father education			
Uneducated	490	0.08	7.8
Primary education		0.63	63.0
Secondary education		0.24	23.7
College/University education		0.06	5.5
Parental occupation			
Farmers	490	0.44	43.5
Non-farmers		0.56	56.5
Difficulty respiratory infection			
Diarrhea illness	490	0.15	14.6
Fever		0.14	14.0
Coughing/Flu Infection		0.35	35.1
None infection		0.36	36.4
Clinic Visit (Yes)	490	0.81	81.5
Clinic Visit (No)		0.07	6.8
Clinic Visit (Rarely)		0.12	11.7
Nutrition Intake (Yes)	490	0.43	42.5
Nutrition Intake (No)		0.21	21.4
Nutrition Intake (Rarely)		0.36	36.0
Drinking boiled water (Yes)	490	0.35	35.1
Drinking boiled water (No)		0.65	64.6

Further results on Table 3 on Health and nutrition-related indicators highlight that, a considerable proportion of children experience illnesses, with coughing or flu infections being the most common (35.1%), followed by diarrhea (14.6%) and fever (14.0%), all of which can negatively affect nutritional status. Although a high percentage of respondents (81.5%) report visiting clinics, the presence of those who rarely (11.7%) or never (6.8%) seek care suggests gaps in consistent healthcare utilization. Nutritional intake practices are also concerning, as only 42.5% report adequate nutrition intake, while the rest either lack it (21.4%) or access it only rarely (36.0%), pointing to food insecurity and poor feeding practices. Additionally, safe water practices are limited, with only 35.1% of households drinking boiled water, exposing many children to waterborne diseases that can worsen malnutrition.

**Figure 2:**
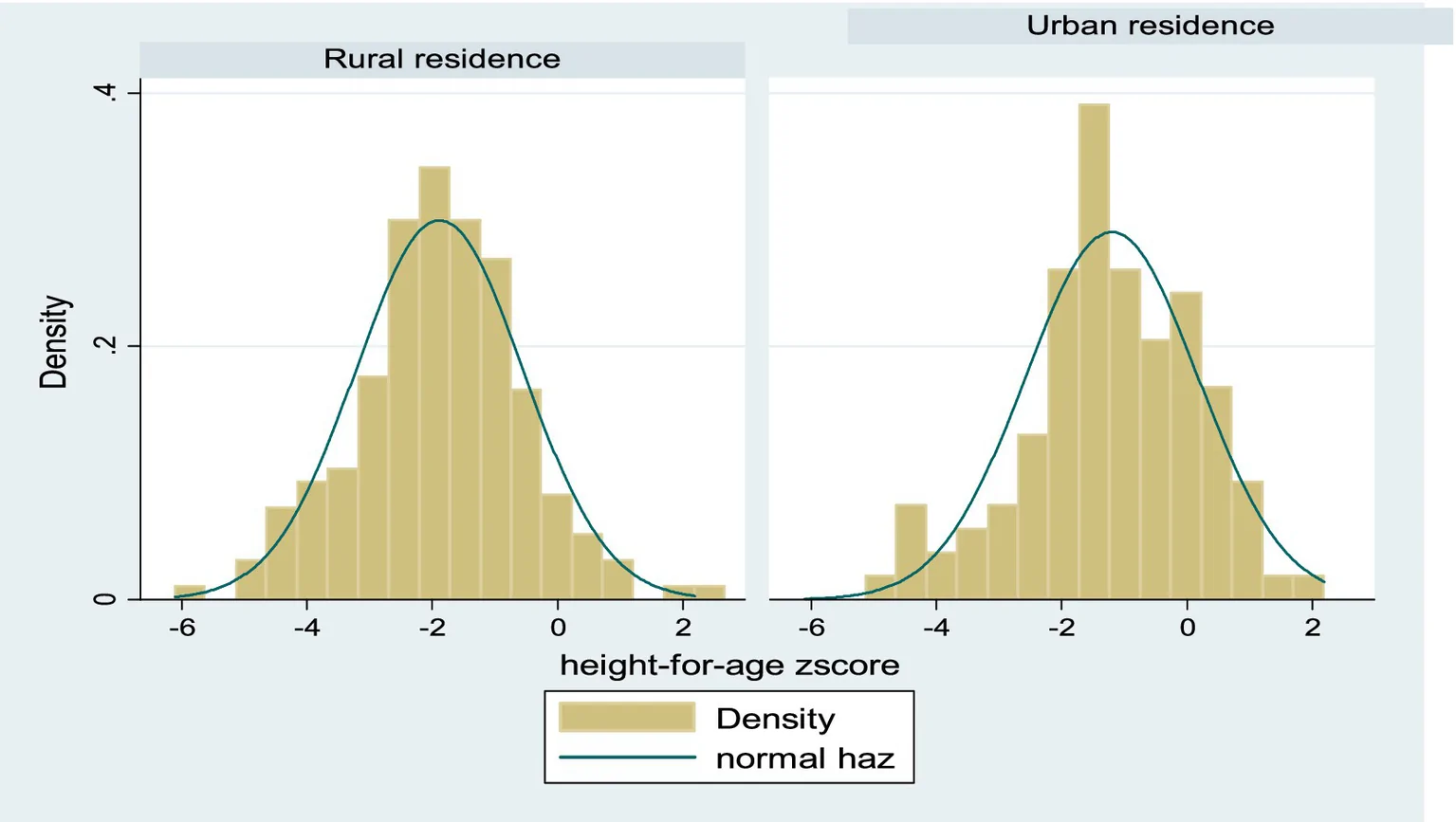
Height for age Z-scores by residences.

Results in Figure 2 show the distribution of height-for-age z-scores for children in rural and urban areas, highlighting clear disparities in nutritional status. In rural areas, the distribution is more heavily shifted to the left, with a larger concentration of children having z-scores below −2, indicating a higher prevalence of stunting and chronic under nutrition. The spread is also wider, suggesting greater inequality in child growth outcomes within rural populations. In contrast, the urban distribution is relatively shifted toward the right, with more children clustered closer to the normal range, although a significant proportion still falls below the recommended standard. The smoother normal curves overlaid on both histograms confirm that while both distributions approximate normality, rural children are systematically disadvantaged. Overall, the figure illustrates that children in urban areas tend to have better growth outcomes than their rural counterparts, reinforcing the existence of rural–urban disparities in child nutrition, likely driven by differences in access to food, healthcare, sanitation, and socioeconomic opportunities.

**Figure 3:**
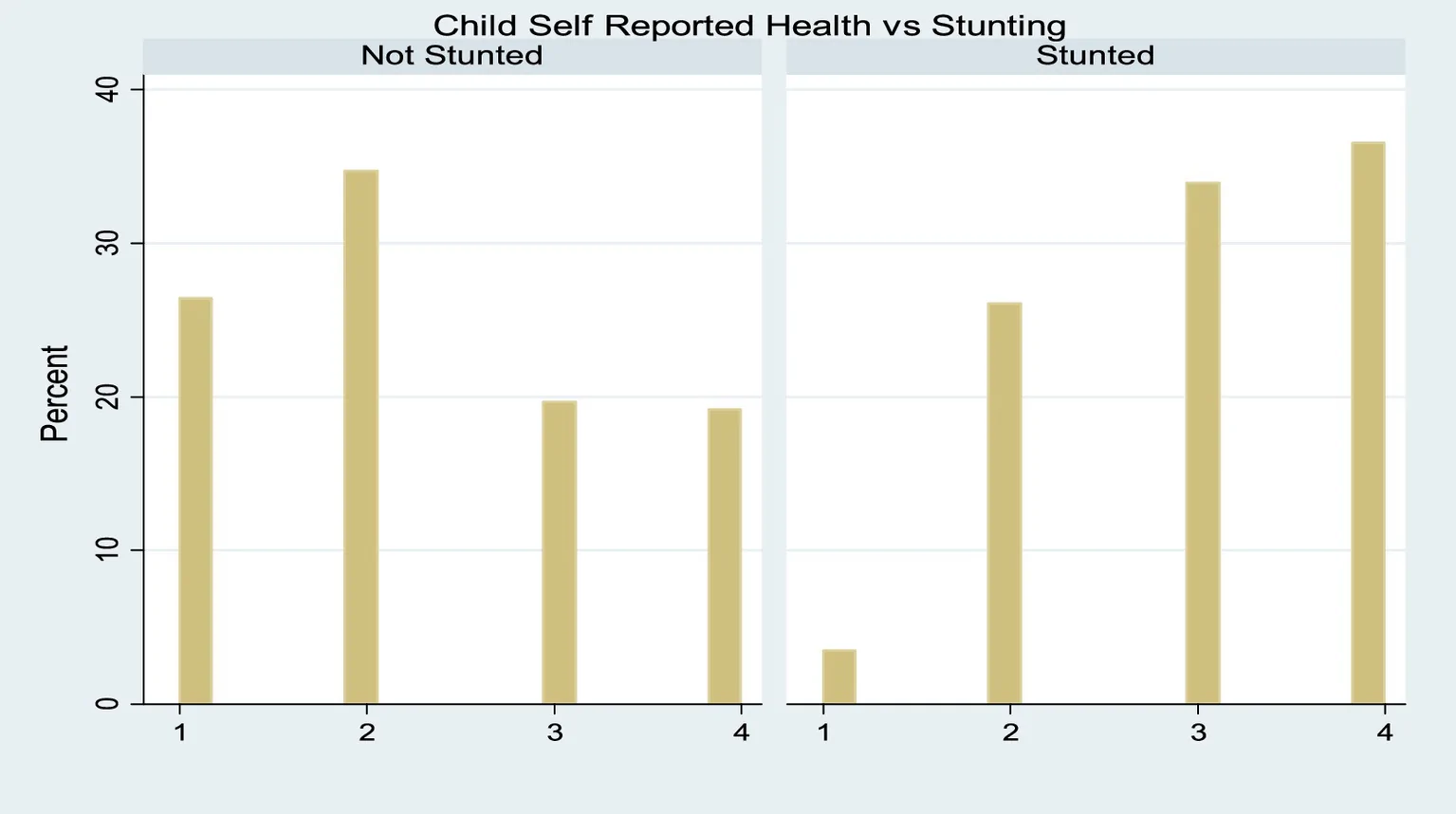
Child self-reported health and stunting.

The results in Figure 3 compare child self-reported health status between stunted and non-stunted children, revealing a clear association between poor growth outcomes and perceived health. Among non-stunted children, the distribution is more balanced, with a higher concentration in the better health categories (particularly levels 2 and 1), indicating that these children are generally perceived to be in relatively good health. In contrast, stunted children are disproportionately concentrated in the poorer health categories (levels 3 and 4), with very few reporting the best health status. This shift toward worse self-reported health among stunted children suggests that chronic under nutrition is closely linked not only to physical growth deficits but also to overall well-being and vulnerability to illness. The pattern reinforces the idea that stunting is a broader indicator of deprivation, reflecting cumulative disadvantages in nutrition, healthcare access, and living conditions. Therefore, this results highlight that children who are stunted are much more likely to experience and report poorer health outcomes compared to their non-stunted counterparts. Further explanations and differences on the same presented at Appendix Table A1.

### 4.2 Effects of household socioeconomic status on child nutritional status

Results in Table 4 presents the estimated effects of socioeconomic factors on child nutritional outcomes (HAZ) across pooled, urban, and rural samples, revealing notable contrasts. Mother’s education shows no statistically significant influence in the pooled sample (primary: 0.095, *p* = 0.686; secondary: −0.131, *p* = 0.641; college: 0.064, *p* = 0.901), and this insignificance persists in both urban (primary: −0.532, *p* = 0.155; secondary: −0.652, *p* = 0.083; college: −0.221, *p* = 0.179) and rural areas (primary: 0.334, *p* = 0.207; secondary: 0.183, *p* = 0.613; college: 1.348, *p* = 0.330), although the negative coefficients in urban areas suggest a contrary pattern where higher maternal education does not necessarily translate into better child nutrition. In contrast, father’s education shows stronger effects, particularly in the pooled sample where primary education is positive and significant (0.642, *p* = 0.023), while secondary (0.566, *p* = 0.074) and college levels (0.749, *p* = 0.089) are marginally significant. However, results in urban areas are contrary to this, showing weaker and insignificant effects (primary: 0.208, *p* = 0.195; secondary: 0.174, *p* = 0.216; college: 0.216, *p* = 0.115), whereas rural results partially align with pooled findings, with primary education nearly significant (0.621, *p* = 0.053) and secondary (0.644, *p* = 0.102) showing moderate positive influence.

**Table 4:** Effects of socioeconomic status on child nutritional outcome (HAZ).

Variables	Pooled data		Urban residences		Rural residences	
	Coefficient	*p*-values	Coefficient	*p*-values	Coefficient	*p*-values
Mother education						
Primary level	0.095	0.686	−0.532	0.155	0.334	0.207
Secondary level	−0.131	0.641	−0.652	0.083	0.183	0.613
College/university	0.064	0.901	−0.221	0.179	1.348	0.330
Father education						
Primary level	0.642	0.023	0.208	0.195	0.621	0.053
Secondary level	0.566	0.074	0.174	0.216	0.644	0.102
College/university level	0.749	0.089	0.216	0.115	0.384	0.657
Parental occupation						
Farmers	0.083	0.622	0.210	0.525	0.257	0.296
Difficulty respiratory infection						
Fever	−0.050	0.855	−0.038	0.916	−0.104	0.768
Coughing/Flu Infection	−0.410	0.075	−0.230	0.467	−0.523	0.095
None infection	0.076	0.744	0.523	0.113	−0.174	0.581
Clinic visits						
No clinic visits	−0.147	0.620	−0.128	0.736	−0.315	0.378
Rarely clinic visits	−0.336	0.146	−0.582	0.158	−0.232	0.443
Nutrition intake						
No nutrition intake	−0.758	0.000	−1.676	0.002	−0.407	0.112
Rarely nutrition intake	−0.280	0.124	−0.659	0.013	−0.044	0.861
Drinking boiled water						
No drinking boiled water	−0.448	0.015	−0.342	0.191	−0.499	0.053
Log of household size	0.011	0.957	0.148	0.584	0.042	0.878
Log of Income	0.167	0.019	0.154	0.200	0.098	0.281
Sex of a child						
Female	0.399	0.007	0.413	0.078	0.420	0.031
R-squared	0.2320		0.3920		0.1460	
Number of Observations	490		193		297	

<0.01 significant at 1%; <0.05 significant at 5%; < 0.1 significant at 10%.

Parental occupation (farmer) is not significant across all samples, though it remains positive (pooled: 0.083, *p* = 0.622; urban: 0.210, *p* = 0.525; rural: 0.257, *p* = 0.296), suggesting limited direct influence of farming on child nutrition.

Regarding health conditions, fever shows negligible and insignificant negative effects across pooled (−0.050, *p* = 0.855), urban (−0.038, *p* = 0.916), and rural (−0.104, *p* = 0.768) samples. Coughing/flu infection presents a negative association in all cases, with marginal significance in pooled (−0.410, *p* = 0.075) and rural (−0.523, *p* = 0.095) samples, while urban results are weaker (−0.230, *p* = 0.467), indicating that illness may matter more in rural settings. Interestingly, having no infection shows a positive but insignificant effect in pooled (0.076, *p* = 0.744) and urban (0.523, *p* = 0.113) samples, but a negative coefficient in rural areas (−0.174, *p* = 0.581), suggesting inconsistent health dynamics. Clinic visits also show no strong significance, although coefficients are consistently negative for limited access (pooled: no visits −0.147, *p* = 0.620; rarely −0.336, *p* = 0.146; urban: −0.128, *p* = 0.736 and −0.582, *p* = 0.158; rural: −0.315, *p* = 0.378 and −0.232, *p* = 0.443), implying that reduced healthcare utilization may worsen nutrition, though evidence is weak.

Nutrition intake stands out as a key determinant with strong and consistent effects. In the pooled sample, no nutrition intake has a large negative and highly significant effect (−0.758, *p* = 0.000), and this effect is even stronger in urban areas (−1.676, *p* = 0.002), showing that lack of food access severely reduces HAZ. Results in urban areas are consistent with pooled findings, as rarely having nutrition is also significant (−0.659, *p* = 0.013), while in rural areas, results are contrary, showing weaker and insignificant effects (no intake: −0.407, *p* = 0.112; rarely: −0.044, *p* = 0.861). Drinking unboiled water is negatively associated with HAZ in the pooled sample (−0.448, *p* = 0.015), but results in urban areas are contrary, showing insignificance (−0.342, *p* = 0.191), while rural results are marginally significant (−0.499, *p* = 0.053), highlighting the stronger role of sanitation challenges in rural setting.

Household characteristics further illustrate disparities across settings. Household size has no meaningful effect across pooled (0.011, *p* = 0.957), urban (0.148, *p* = 0.584), and rural (0.042, *p* = 0.878) samples. In contrast, income positively and significantly affects HAZ in the pooled sample (0.167, *p* = 0.019), but results in urban (0.154, *p* = 0.200) and rural (0.098, *p* = 0.281) areas are contrary, showing no significant effect when disaggregated. Gender differences are evident, with female children having higher HAZ in pooled (0.399, *p* = 0.007) and rural samples (0.420, *p* = 0.031), while urban results are weaker and only marginally significant (0.413, *p* = 0.078). Overall, the explanatory power of the model is higher in urban areas (R^2^ = 0.392) compared to pooled (R^2^ = 0.232) and especially rural areas (R^2^ = 0.146), suggesting that observed variables better capture nutritional determinants in urban settings, whereas rural child nutrition is influenced by additional unobserved factors.

### 4.3 Instrumentation on factors influecning child nutrition (HAZ)

Table 5 presents the Two Stage Least Squares (2SLS) results on the determinants of child nutrition (HAZ), using distance to the nearest health facility as an instrument, and reveals notable differences across pooled, urban, and rural samples. The coefficient on log of income is positive in the pooled (0.1221) and rural (0.0938) samples but negative in urban areas (−0.0452); however, in all cases it is statistically insignificant, suggesting that income does not play a decisive role in explaining child nutrition once endogeneity is controlled for. Contrary to expectations that higher income should improve nutrition, these findings imply that non-income factors may be more binding constraints. In contrast, the sex of the child shows a consistent positive effect for females across all samples, with statistical significance in the pooled (*p* = 0.004) and rural (*p* = 0.024) data, but only marginal significance in urban areas (*p* = 0.076). This indicates that female children tend to have better nutritional outcomes than males, although the strength of this relationship appears weaker in urban settings.

**Table 5:** Two stage least squares (2SLS) results on determinants of child nutrition (HAZ).

Variables	Pooled data		Urban residence		Rural residence	
HAZ	Coef.	*P* > z	Coef.	*P* > z	Coef.	*P* > z
Log of income	0.1221	0.476	−0.0452	0.886	0.0938	0.647
Sex of a child						
Female	0.4161	0.004	0.3917	0.076	0.4215	0.024
Log of household size	−0.0078	0.969	0.2777	0.416	0.0196	0.941
Drinking boiled water						
No	−0.4522	0.018	−0.4484	0.139	−0.4966	0.048
Household occupation						
Farmers	0.0748	0.722	0.1709	0.569	0.2652	0.322
Clinic visits						
No	−0.1896	0.527	−0.1311	0.814	−0.2919	0.407
Rarely	−0.3595	0.114	−0.5598	0.113	−0.2522	0.391
Nutrition intake						
No	−0.7804	0.000	−1.7546	0.000	−0.4301	0.088
Rarely	−0.3432	0.053	−0.7196	0.013	−0.1249	0.602
Chronic illness						
Fever	−0.0571	0.838	−0.1496	0.731	0.0002	0.999
Coughing/flu infections	−0.3519	0.121	−0.1658	0.617	−0.4112	0.172
None	0.1414	0.564	0.7056	0.086	−0.0631	0.840
R-squared	0.2153		0.3535		0.1148	
Number of observations	490		193		297	

<0.01 significant at 1%; <0.05 significant at 5%; < 0.1 significant at 10%.

Household-level characteristics present mixed and largely insignificant effects across the three samples. The log of household size is negative in the pooled data (−0.0078) but positive in both urban (0.2777) and rural (0.0196) samples; however, none of these coefficients are statistically significant, suggesting no clear relationship between household size and child nutrition. Similarly, household occupation (being farmers) shows positive coefficients across pooled, urban, and rural samples, yet remains statistically insignificant throughout, implying that occupation type does not strongly differentiate nutritional outcomes. However, drinking unboiled water exhibits a consistently negative effect across all samples, and is statistically significant in the pooled (*p* = 0.018) and rural (*p* = 0.048) results, but not in urban areas (*p* = 0.139). This suggests that unsafe water consumption is a critical determinant of poor child nutrition, particularly in rural areas where water quality challenges are more pronounced, whereas urban households may have relatively better access to safer water sources.

Behavioral and health service utilization variables reveal further contrasts. Clinic visits, whether “No” or “Rarely,” show negative coefficients across all samples; however, none are statistically significant, indicating that frequency of clinic visits does not have a measurable impact on child nutrition in this model. This is somewhat surprising and may reflect issues related to the quality or effectiveness of healthcare services rather than access alone. In contrast, nutrition intake emerges as one of the most robust determinants. Children with no nutrition intake exhibit large negative and highly significant effects in the pooled (*p* = 0.000) and urban (*p* = 0.000) samples, and a weaker but still negative effect in rural areas (*p* = 0.088). Likewise, rarely having adequate nutrition intake is negatively associated with HAZ, with significance in urban areas (*p* = 0.013) and marginal significance in the pooled sample (*p* = 0.053), but not in rural areas. Thus, while inadequate nutrition consistently reduces child health outcomes, its statistical strength is more pronounced in urban contexts compared to rural ones. Finally, chronic illness variables show generally weak and inconsistent effects across all samples. Fever and coughing/flu infections have negative coefficients in most cases, while the category “None” is positive in pooled and urban samples but negative in rural areas; however, none of these variables are statistically significant except for a marginal effect of “None” in urban areas (*p* = 0.086). Generally, the results highlight that, while income and healthcare utilization appear less influential, factors such as nutrition intake, water safety, and gender differences are more critical, with varying importance across urban and rural setting.

### 4.4 Linkage between household socioeconomic variables and child health

Table 6 presents the Structural Equation Model (SEM) results linking key household socioeconomic and intervening variables to child nutrition (HAZ) and self-reported health (SRH) across pooled, urban, and rural samples, revealing both consistent patterns and notable contrasts. For all residences combined, nutrition intake has a negative and statistically significant association with HAZ (*p* = 0.015) but a positive and significant effect on SRH (*p* = 0.003), suggesting that while inadequate nutrition intake worsens anthropometric outcomes, improvements in intake are strongly associated with better perceived health status. Similarly, clinic visits show a positive and significant effect on SRH (*p* = 0.002), however, contrary to expectations, they are negatively associated with HAZ and not statistically significant (*p* = 0.234), implying that healthcare utilization may respond to illness rather than prevent poor nutrition outcomes. Drinking boiled water shows a negative and statistically significant association with HAZ (*p* = 0.000), which appears counterintuitive. This finding may reflect reverse causality, where households with undernourished or frequently ill children are more likely to adopt water-boiling practices as a protective response. It may also indicate unobserved environmental or household factors, such as poor sanitation or unsafe water sources, that were not fully captured in the SEM model. In contrast, the relationship between drinking boiled water and SRH is positive but statistically insignificant

**Table 6:** Household socioeconomic indicators and intervening variables in SEM Analysis.

Variables	HAZ		SRH	
	Coefficient	*P*-values	Coefficient	*P*-values
All residences				
Nutrition Intake	−0.2207	0.015	0.2111	0.003
Clinic Visits	−0.1354	0.234	0.3990	0.002
Drinking Boiled water	−0.7234	0.000	0.0781	0.375
Urban				
Nutrition Intake	−0.4124	0.002	0.2169	0.053
Clinic Visits	−0.2968	0.110	0.3258	0.038
Drinking Boiled water	−0.5132	0.046	−0.1339	0.538
Rural				
Nutrition Intake	−0.0831	0.485	0.1297	0.209
Clinic Visits	−0.0823	0.558	−0.0959	0.431
Drinking Boiled water	−0.6036	0.010	0.3679	0.071

<0.01 significant at 1%; <0.05 significant at 5%; < 0.1 significant at 10%.

Disaggregating by residence highlights important differences. In urban areas, nutrition intake remains a strong determinant, with a larger negative and highly significant effect on HAZ (*p* = 0.002) and a marginally significant positive effect on SRH (*p* = 0.053), indicating that dietary factors are particularly influential in cities. Clinic visits, however, show a positive and significant impact on SRH (*p* = 0.038), contrary to their insignificant role in HAZ, reinforcing the idea that healthcare improves perceived health more than physical growth outcomes. Drinking boiled water is negatively and significantly associated with HAZ (*p* = 0.046) but remains insignificant for SRH. In contrast, rural results show a weakening of most relationships: nutrition intake and clinic visits are both insignificant for HAZ and SRH, suggesting that other unobserved constraints dominate in rural settings. However, drinking boiled water retains a negative and significant effect on HAZ (*p* = 0.010) and a positive, marginally significant effect on SRH (*p* = 0.071), highlighting its continued importance for health in rural areas. Overall, the findings suggest that while nutrition intake and healthcare utilization are more influential in urban settings, water safety plays a consistently important role, particularly in rural areas, and perceived health (SRH) responds more strongly to these variables than objective nutrition outcomes (HAZ).

**Figure 4:**
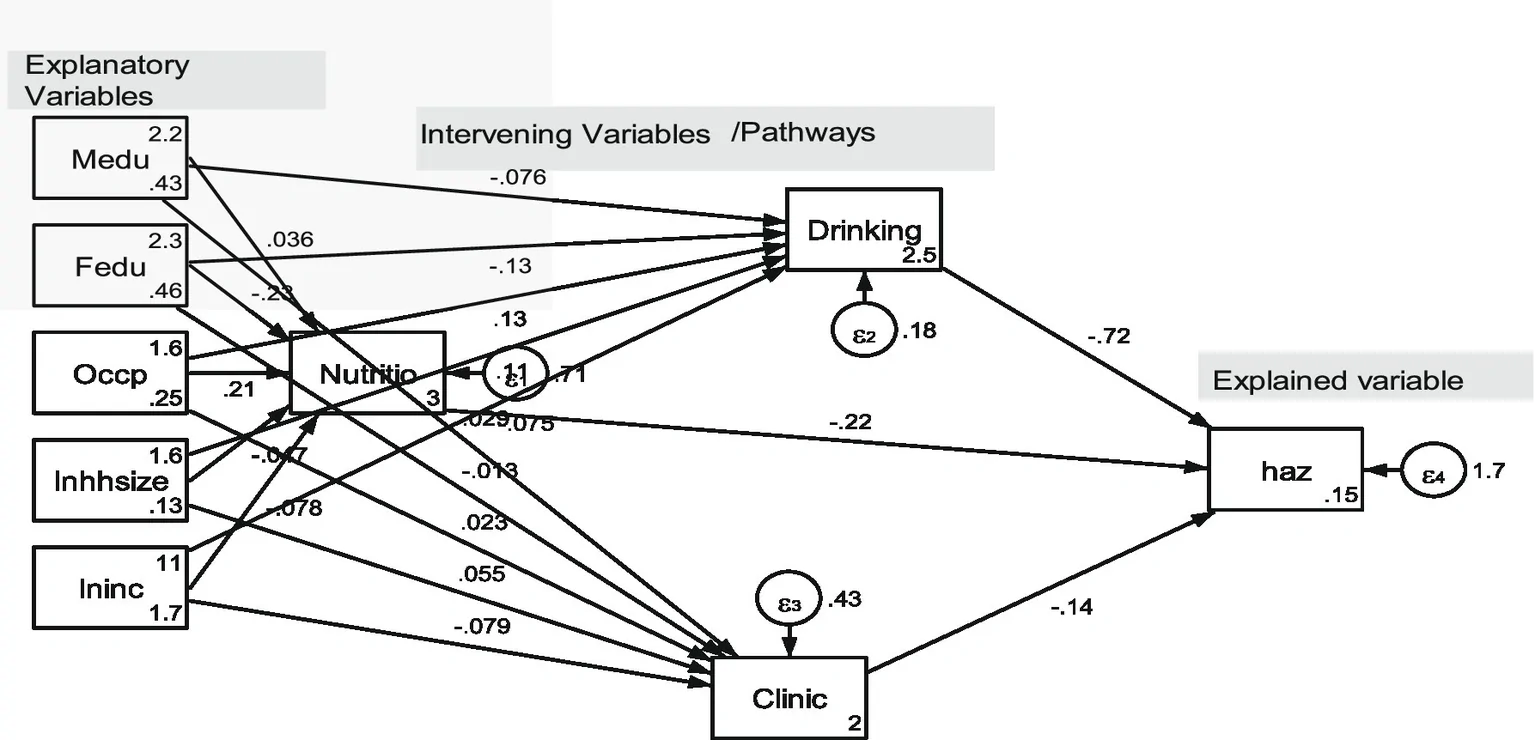
Relationship between socioeconomic variable and health outcomes (child nutritional status-HAZ).

Moreover, results in Figure 4 presents the pathways through which parental education, occupation, household size, and income influence child nutritional outcomes via intervening behaviors such as nutrition intake, drinking boiled water, and clinic visits. The coefficients indicate that nutrition intake and drinking boiled water exert the strongest negative effects on child health status (HAZ), with values of −0.22 and −0.72 respectively, while clinic visits show a weaker and statistically insignificant effect (−0.14). Positive associations are observed between clinic visits and nutrition intake (0.43) as well as between drinking boiled water and nutrition intake (0.22), suggesting that these behaviors reinforce each other. Overall, the model highlights that socioeconomic factors shape household practices, which in turn significantly determine child health outcomes, with nutrition intake emerging as the most consistent mediating pathway.

**Figure 5:**
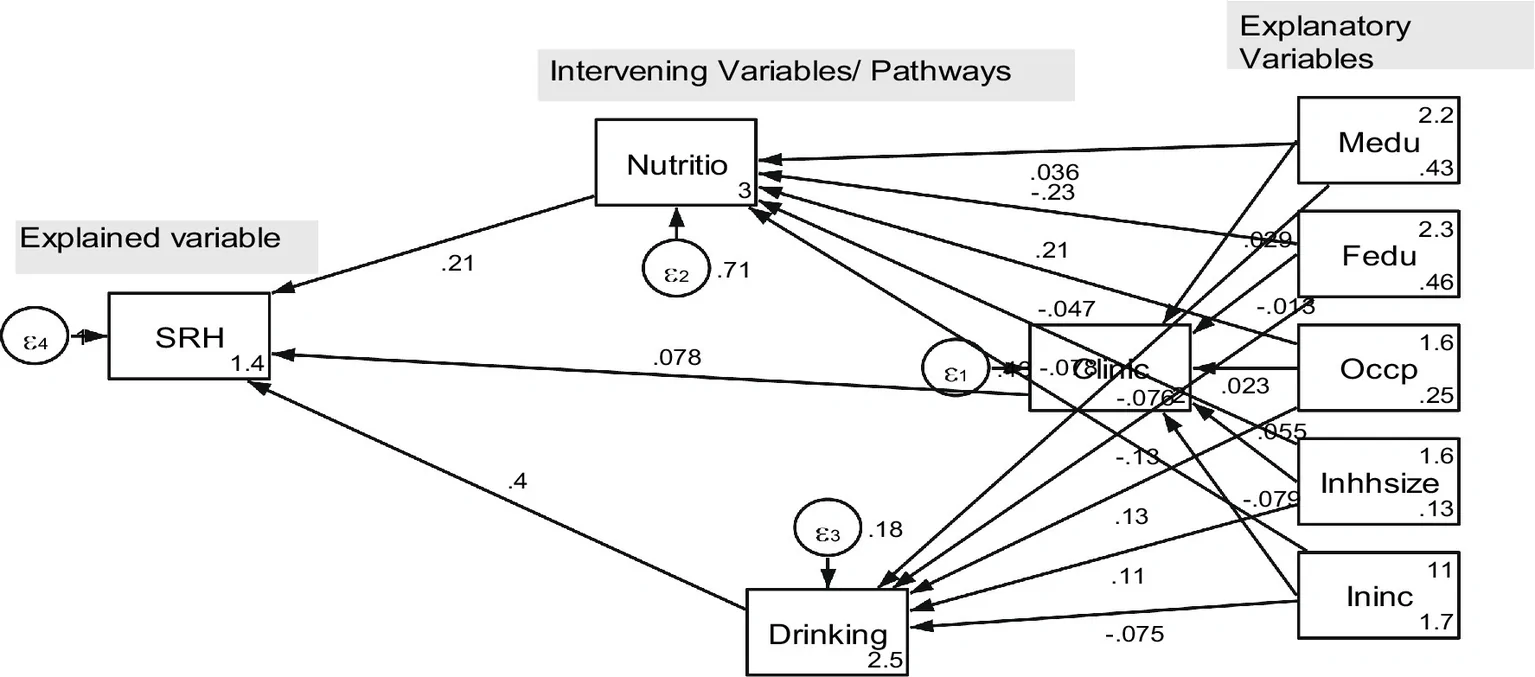
Relationship between household socioeconomic variables, intervening variables (pathways) and SRH.

Results in Figure 5 presents the pathways through which socioeconomic characteristics such as parental education, occupation, household size, and income indirectly shape self-rated health (SRH) through household practices like nutrition intake and drinking boiled water. The model indicates that SRH is positively associated with both nutrition intake (0.078) and drinking boiled water (0.4), highlighting the importance of these behaviors in improving perceived health outcomes. Moreover, nutrition intake and drinking boiled water are themselves influenced by parental education and occupation, with mixed directions of effect as maternal and paternal education showing both positive and negative coefficients, while occupation and household size exert smaller influences. Notably, clinic visits also emerge as a reinforcing pathway, positively linked to nutrition intake (0.21) and drinking boiled water (0.4), suggesting that health-seeking behavior strengthens household nutrition practices. Thus, these results emphasize that SRH is not only a direct outcome of individual behaviors but also a reflection of broader socioeconomic conditions that shape household pathways, with nutrition intake acting as a central mediating factor.

The diagnostic test for Structural Equation Model (SEM) on Table 7 demonstrates an excellent overall fit to the data. The default model estimated 26 free parameters and produced a non-significant chi-square statistic (*χ*^2^ = 21.864, df = 24, *p* = 0.594), indicating that the model does not significantly differ from the observed data. The chi-square to degrees of freedom ratio (*χ*^2^/df = 0.911) is well below the recommended threshold of 3.0, further confirming good model fit. Incremental fit indices also show strong performance, with NFI = 0.957, TLI = 1.006, IFI = 1.000, and CFI = 1.000, all exceeding conventional cut-off values. In addition, the absolute fit measures are highly satisfactory, with low residual error (RMR = 0.011), high goodness-of-fit values (GFI = 0.989; AGFI = 0.972), and an RMSEA of 0.000, indicating no meaningful approximation error. Therefore, these diagnostic statistics suggest that the specified SEM is well fitted, parsimonious, and free from major specification problems.

**Table 7:** Summarized diagnostic test for structural equation modelling.

Summary	Model	NPAR	CMIN (*χ*^2^)	DF	*P*	CMIN/DF (*χ*^2^/DF)
Chi square minimum (CMIN)	Default model	26	21.864	24	0.594	0.911
	Saturated model	45	0.000	0	–	–
	Independence model	9	512.447	36	0.000	14.235

	Model	NFI (Delta1)	RFI (rho1)	IFI (Delta2)	TLI (rho2)	CI
The Baseline Comparison test	Default model	0.957	0.938	1.000	1.006	1.000
	Saturated model	1.000	1.000	1.000	1.000	1.000
	Independence model	0.000	0.000	0.000	0.000	0.000

	Model	RMSEA	LO 90	HI 90	PCLOSE
Root mean square residual (RMR)	Default model	0.000	0.000	0.041	0.873
	Saturated model	0.000	0.000	0.000	1.000
	Independence model	0.287	0.266	0.309	0.000

	Model	ECVI	LO 90	HI 90	MECVI
The Expected Cross-Validation Index (ECVI)	Default model	0.176	0.169	0.228	0.191
	Saturated model	0.201	0.201	0.201	0.201
	Independence model	4.938	4.512	5.467	5.011

	Model	PRATIO	PNFI	PCFI
Parsimony adjusted measures	Default model	0.667	0.638	0.667
	Saturated model	0.000	0.000	0.000
	Independence model	1.000	0.000	0.000

### 4.5 Estimation of rural–urban child health gap

Table 8 presents the Blinder–Oaxaca decomposition results of child nutrition (HAZ), comparing rural (group 1) and urban (group 2) children and separating the observed nutrition gap into explained and unexplained components. The results show that rural children have significantly lower nutritional status, with a mean HAZ of −1.886 compared to −1.200 for urban children, and the overall difference (gap) of −0.685 is statistically significant (*p* = 0.000), indicating a clear and meaningful urban–rural disparity in child nutrition. The decomposition further reveals that the “explained” component of −0.491 is highly significant (p = 0.000), implying that a substantial share of the nutrition gap is driven by differences in observed characteristics between rural and urban households, such as socioeconomic conditions, access to services, and behavioral factors. In contrast, the “unexplained” component of −0.194 is only marginally significant (*p* = 0.093), suggesting that differences in returns to characteristics or unobserved factors such as discrimination, structural inefficiencies, or unmeasured heterogeneity play a relatively smaller role in explaining the nutrition gap.

**Table 8:** Blinder-Oaxaca decomposition results using HAZ as an outcome.

HAZ	Coefficient	Robust std. err.	*P*-value
Overall estimation			
group_1 (Rural)	−1.8860	0.094	0.000
group_2 (Urban)	−1.2000	0.130	0.000
Difference (Gap)	−0.6850	0.161	0.000
Explained	−0.4910	0.119	0.000
Unexplained	−0.1940	0.115	0.093
Number of Observation	490		

<0.01 significant at 1%; <0.05 significant at 5%; < 0.1 significant at 10%.

Table 9 presents the Blinder–Oaxaca decomposition of the rural–urban HAZ gap by breaking down the “explained” component into the contribution of specific household and child-level characteristics. Overall, the explained gap remains negative and statistically significant (−0.491, *p* = 0.000), confirming that observable differences between rural and urban households account for a substantial share of the nutrition disparity. Among socioeconomic variables, most maternal education categories (no education, primary, and secondary) show small and statistically insignificant contributions, indicating that maternal education differences between rural and urban areas do not strongly drive the HAZ gap in this sample. Similarly, paternal education mostly shows insignificant effects, except for fathers with no education, which has a negative and statistically significant contribution (−0.079, *p* = 0.048), suggesting that differences in paternal education disadvantage rural children relative to urban ones. Parental occupation and household size also contribute negligibly and are statistically insignificant, implying limited explanatory power in accounting for the rural–urban nutrition divide.

**Table 9:** Contribution of the explanatory variables to the explained rural–urban gap.

HAZ	Coefficient	Robust std. err.	*P-*value
Mother with no education	0.003	0.049	0.948
Mother’s primary education	0.018	0.062	0.772
Mother’s secondary education	0.030	0.086	0.728
Father with no education	−0.079	0.040	0.048
Father’s primary education	−0.040	0.078	0.607
Father’s secondary education	0.058	0.071	0.415
Parental occupation	0.032	0.092	0.724
Diarrhea illness	0.003	0.009	0.730
Fever illness	−0.007	0.015	0.644
Coughing/flu infection	−0.024	0.028	0.397
Nutrition intake (Yes)	−0.069	0.044	0.118
Nutrition intake (No)	0.099	0.008	0.038
Drinking boiled water	−0.142	0.058	0.014
Clinic visit (Yes)	−0.009	0.014	0.53
Clinic visit (No)	0.006	0.013	0.649
Log of Household income	−0.181	0.078	0.021
Log of Household size	0.009	0.038	0.821
Explained	−0.491	0.119	0.000
Unexplained	−0.194	0.115	0.093
Number of Observation	490		

<0.01 significant at 1%; <0.05 significant at 5%; < 0.1 significant at 10%.

In contrast, child health and behavioral factors play a more meaningful role in explaining the gap. Notably, nutrition intake variables show mixed but important effects: children with no nutrition intake contribute positively and significantly to the explained gap (0.099, *p* = 0.038), indicating that higher prevalence of inadequate nutrition in rural areas is a key driver of poorer HAZ outcomes. Conversely, the “nutrition intake (Yes)” category is negative but not significant, suggesting weaker contribution when adequate nutrition is present. Drinking boiled water emerges as one of the most important determinants, with a large negative and statistically significant coefficient (−0.142, *p* = 0.014), indicating that disparities in water safety practices between rural and urban households significantly widen the nutrition gap. Similarly, household income is a significant contributor (−0.181, *p* = 0.021), confirming that income differences remain an important structural driver of rural disadvantage, even if income was insignificant in earlier regression models.

Finally, health status and service utilization variables contribute relatively little to explaining the gap. Diarrhea, fever, and coughing/flu infections all show small and statistically insignificant effects, implying that differences in reported illness do not meaningfully explain rural–urban disparities in child nutrition. Likewise, clinic visits (both yes and no categories) are insignificant, suggesting that differences in healthcare utilization patterns do not significantly contribute to the explained gap. Generally, the results indicate that the rural–urban HAZ disparity is primarily driven by differences in income, drinking water practices, and nutrition intake, while education, occupation, health service use, and most illness variables play a limited role. This implies that policy efforts aimed at reducing child nutrition inequality should prioritize improving household economic conditions, dietary practices, and access to safe drinking water, particularly in rural areas where these disadvantages are more pronounced.

#### 4.5.1 Fairlie non-linear decomposition results

Table 10 presents the Fairlie non-linear decomposition of the rural–urban gap in self-reported health (SRH), breaking down how observable characteristics contribute to the overall health disparity. The results show that urban residents report significantly better health outcomes, with a predicted SRH of 0.555 compared to 0.434 for rural residents, producing a negative rural–urban gap of −0.120. The explained portion of this gap is −0.091, indicating that most of the observed difference in self-reported health between rural and urban populations is attributable to differences in characteristics rather than unobserved factors. Among socioeconomic variables, most parental education categories (mother’s no education, father’s no education, father’s primary education) are statistically insignificant, suggesting that education differences between rural and urban households do not strongly drive disparities in perceived health. Similarly, parental occupation also has an insignificant contribution, reinforcing the idea that structural employment differences play a limited role in explaining SRH inequality.

**Table 10:** Fairlie non-linear decomposition results with the contribution of the observed characteristics to the explained rural–urban gap.

Observed characteristics	Coefficient	Std. error	*p*-value
Mother with no education	0.003	0.015	0.821
Mother’s primary education	0.022	0.012	0.066
Father with no education	−0.006	0.013	0.663
Father’s primary education	−0.026	0.026	0.318
Parental occupation	−0.034	0.048	0.480
Diarrhea illness	0.004	0.004	0.313
Fever illness	0.002	0.005	0.748
Coughing/flu infection	0.000	0.005	0.951
Nutrition intake (Yes)	−0.041	0.021	0.052
Nutrition intake (No)	−0.033	0.016	0.045
Drinking boiled water	−0.016	0.031	0.605
Clinic visit (Yes)	−0.006	0.006	0.283
Clinic visit (No)	0.004	0.006	0.478
Log of Household income	0.052	0.037	0.155
Log of Household size	−0.025	0.016	0.133
SRH	Predicted values		
Predicted value in rural residence		0.434	
Predicted value in urban residence		0.555	
Difference (rural–urban gap)		−0.120	
Total explained gap		−0.091	

<0.01 significant at 1%; <0.05 significant at 5%; < 0.1 significant at 10%.

In contrast, nutrition-related variables emerge as the most important contributors to the rural–urban health gap. Nutrition intake shows a significant and meaningful contribution, where “nutrition intake (No)” is negative and statistically significant (−0.033, *p* = 0.045), indicating that inadequate nutrition in rural areas reduces self-reported health and is a key driver of the observed disparity. The “nutrition intake (Yes)” variable is also marginally significant (−0.041, *p* = 0.052), reinforcing the central role of dietary quality in shaping perceived health outcomes. This suggests that differences in dietary adequacy between rural and urban households are one of the most important observable factors explaining why rural residents report poorer health. However, contrary to expectations, drinking boiled water, clinic visits (both yes and no), and most illness variables (diarrhea, fever, and coughing/flu infections) are statistically insignificant, implying that differences in healthcare utilization and reported illness conditions do not meaningfully explain the rural–urban SRH gap in this decomposition framework.

Finally, economic and demographic factors also show limited explanatory power. Household income is positive but insignificant (0.052, *p* = 0.155), suggesting that although urban households are generally wealthier, income differences alone do not significantly account for differences in perceived health status. Likewise, household size is negative but insignificant (−0.025, *p* = 0.133), indicating minimal contribution to the observed gap. Overall, the Fairlie decomposition results indicate that the rural–urban disparity in self-reported health is driven primarily by differences in nutrition-related behaviors, while education, income, occupation, health service utilization, and illness conditions contribute relatively little. This implies that improving dietary adequacy in rural areas would likely yield the greatest reduction in perceived health inequalities between rural and urban populations. For additional insights and detailed results (see Appendix Table A3).

## 5 Discussion

### 5.1 Household socioeconomic status and nutritional status

The findings on household socioeconomic status reveal a complex and sometimes contradictory relationship with child nutritional outcomes. Parental education, particularly father’s education, emerges as an important determinant of child HAZ, consistent with the human capital theory embedded in the Grossman health production model, which emphasizes that education improves health knowledge, decision-making, and resource allocation within households (17, 35). Specifically, children whose fathers attained primary education exhibit better nutritional outcomes, supporting the argument that educated parents are more likely to invest in preventive health behaviors and appropriate feeding practices (36). However, contrary to expectations and much of the existing literature, maternal education in urban areas shows a negative association with child nutritional status. This counterintuitive finding may reflect opportunity costs and time constraints faced by educated mothers in urban labor markets, where increased employment may reduce time allocated to childcare and feeding practices. Such a result suggests that maternal education alone is not sufficient to guarantee better child health outcomes without supportive childcare environments and policies.

Household income also plays a significant but context-dependent role in shaping child nutrition outcomes. The positive and statistically significant association between income and HAZ indicates that higher-income households are better able to purchase nutritious food, access healthcare, and invest in improved living conditions (39). However, the effect is more pronounced in urban settings than in rural ones, highlighting structural inequalities in access to health inputs across space. In rural areas, even higher income may not fully translate into improved nutrition due to limited availability of diverse foods and healthcare services. Contrary to theoretical expectations, parental occupation does not show a statistically significant effect, suggesting that its influence may operate indirectly through income or other unobserved pathways rather than directly affecting child nutrition. Additionally, the consistent finding that female children exhibit higher HAZ scores aligns with evidence suggesting differential intrahousehold resource allocation, where girls may receive more consistent caregiving or feeding attention in some settings (40).

### 5.2 Linkage between household socioeconomic variables, intervening variables (pathways) and child health

The analysis of intervening variables highlights the importance of behavioral and environmental pathways through which socioeconomic status influences child health, consistent with Grossman’s model of health production. Across households, nutrition intake, clinic visits, and drinking boiled water emerge as key transmission channels linking socioeconomic conditions to child health outcomes. Nutrition intake is particularly influential, reinforcing the idea that dietary quality is a direct mechanism through which household resources translate into improved child growth and perceived health status. Clinic visits also play an important role, especially in influencing self-reported health, suggesting that healthcare utilization improves health awareness and perceived well-being, even if its effect on objective nutrition outcomes is weaker (41). Drinking boiled water further emphasizes the importance of environmental health conditions in preventing infections that undermine nutritional status.

However, the strength of these pathways varies across socioeconomic indicators. Parental occupation, father’s education, and household income are found to be the most influential determinants shaping these intervening behaviors, particularly in relation to nutrition intake and water treatment practices. This suggests that economic capacity and parental knowledge jointly shape health-related decision-making within households. The observed negative associations in some pathways indicate substitution effects, where households with higher income or education may rely less on certain preventive practices, replacing them with other forms of health investment (42). Contrary to several earlier studies that emphasize maternal education as a dominant driver of child health behaviors (43), this study finds limited evidence of such an effect. This discrepancy may be due to the restricted set of intervening variables included, which may not fully capture the pathways through which maternal education influences child health, thereby suggesting the need for broader modeling of behavioral mechanisms.

### 5.3 Socioeconomic status and rural–urban gap in child health

The results clearly demonstrate a persistent and statistically significant rural–urban gap in child health outcomes, with urban children exhibiting better nutritional status than their rural counterparts. This finding is consistent with the fundamental cause theory, which argues that socioeconomic advantage systematically translates into better health outcomes due to improved access to flexible resources such as knowledge, money, and healthcare infrastructure (3, 24). The observed gap in HAZ indicates that structural inequalities between rural and urban settings remain a key driver of child health disparities. Similar patterns have been documented in other contexts, although the magnitude of the gap varies depending on regional socioeconomic conditions, sample composition, and measurement differences across studies (18, 27).

The decomposition analysis further reveals that the majority of the rural–urban health gap is explained by observable characteristics, particularly household income, nutrition intake, and drinking water practices. These findings suggest that improving material living conditions and health-related behaviors in rural areas could substantially reduce the observed disparities. Notably, drinking boiled water and nutrition intake emerge as the most important contributors, reinforcing the idea that environmental and behavioral factors are central to child health inequality. In contrast, parental education contributes less than expected, which contradicts the fundamental cause theory and suggests that its effect may be mediated through other variables or diluted in the presence of stronger socioeconomic constraints (44–46).

The unexplained component of the gap, although smaller, remains statistically significant and points to the presence of unobserved factors such as differences in healthcare quality, cultural practices, or potential structural inefficiencies in service delivery. This residual gap also raises the possibility of discrimination or unequal returns to endowments between rural and urban populations. Furthermore, the analysis of self-reported health reinforces these findings, showing that nutrition intake accounts for a substantial share of the explained gap, while maternal education contributes moderately to perceived health differences. These results align with the broader literature emphasizing that socioeconomic disparities primarily drive health inequalities, but also highlight that behavioral and environmental factors are critical transmission channels that policy must address directly (47, 48).

## 6 Conclusion and policy recommendations

This study provides strong evidence that child nutrition and health outcomes are shaped by a combination of socioeconomic status, behavioral practices, and environmental conditions, with clear rural–urban disparities. Nutrition intake, safe drinking water, and healthcare utilization emerge as the most consistent determinants of both objective (HAZ) and subjective (SRH) health outcomes. The findings emphasize that while income and education are important, their effects are largely mediated through behavioral pathways such as dietary practices and water safety. The decomposition results further reveal that most of the rural–urban health gap is explained by observable characteristics rather than unobserved factors, indicating that targeted interventions could significantly reduce inequality.

Policy implications from the findings suggest the need for both short-term nutrition-focused interventions and broader structural policies aimed at reducing rural–urban socioeconomic inequalities. In the short term, targeted nutrition interventions should prioritize improving dietary intake and child feeding practices through community-based nutrition programs, school feeding initiatives, maternal nutrition education, and the provision of fortified foods and supplements for vulnerable rural households. Improving water safety should also be an immediate priority through hygiene education, subsidized household water treatment products, and community-level water purification initiatives to reduce exposure to waterborne diseases that adversely affect child nutrition outcomes.

At the structural level, the results highlight household income as a major contributor to the explained rural–urban health gap, indicating the importance of policies that address persistent rural economic disadvantages. Long-term strategies should therefore focus on rural livelihood diversification, targeted cash transfer programs, improved agricultural productivity, and support for small-scale farm and non-farm enterprises to strengthen household purchasing power and access to nutritious food. In addition, sustained investments in rural infrastructure, particularly access to clean and reliable water systems, healthcare services, and market connectivity, are essential for reducing broader inequalities in child health outcomes. Although healthcare access and parental education contribute relatively less to the explained gap, they remain important components of an integrated multisectoral approach aimed at improving child health and nutrition in underserved rural communities

Despite the important contributions of this study, several limitations should be acknowledged. The use of cross-sectional data limits the ability to establish temporal relationships and draw definitive causal conclusions; therefore, the findings should be interpreted as associations rather than causal effects. Although Two-Stage Least Squares (2SLS) was applied to reduce potential endogeneity, some variables such as household income, nutritional intake, and healthcare utilization may still be affected by residual endogeneity or omitted variable bias. In addition, the SEM framework identifies structural relationships but may not fully account for reverse causality or unobserved confounding factors. The reliance on self-reported health (SRH) may also introduce reporting bias and subjective interpretation. Furthermore, important determinants of child health and nutrition, including sanitation conditions, dietary diversity, maternal time allocation, and intra-household resource distribution, were not fully captured in the analysis. Finally, the focus on a single region may limit the generalizability of the findings to other socioeconomic and institutional settings. Future studies should employ longitudinal data and incorporate broader contextual and behavioral variables to strengthen causal interpretation and external validity.

## Data Availability

The original contributions presented in the study are included in the article/Supplementary material, further inquiries can be directed to the corresponding author.
