# Supplementary material for: Rural–urban disparities in child nutrition in Tabora, Tanzania: a socioeconomic decomposition and implications for food security policy
Source: Front Nutr. 2026 Jul 20;13:1800873. doi: 10.3389/fnut.2026.1800873 (PMC13430998; doi:10.3389/fnut.2026.1800873)
Supplement: Supplementary file 1 [file Table_1.docx]

**Table A1: Summary** **statistics on residence, Child self-reported health (SRH) and Stunting (HAZ)**

| **SRH** | | | | **HAZ** | | | |
| --- | --- | --- | --- | --- | --- | --- | --- |
| **SRH** | **Rural** | **Urban** | **Total** |  | **Rural** | **Urban** | **Total** |
| Good Health | 98 | 136 | 234 | Stunted | 191 | 52 | 243 |
| Bad/Ill Health | 211 | 45 | 256 | Not Stunted | 78 | 169 | 247 |
| **Total** | **309** | **181** | **490** | **Total** | **269** | **221** | **490** |
