# Supplementary material for: Rural–urban disparities in child nutrition in Tabora, Tanzania: a socioeconomic decomposition and implications for food security policy
Source: Front Nutr. 2026 Jul 20;13:1800873. doi: 10.3389/fnut.2026.1800873 (PMC13430998; doi:10.3389/fnut.2026.1800873)
Supplement: Supplementary file 2 [file Table_2.docx]

**Table A2: Linkage between household socioeconomic variables, intervening variables (pathways) and child health**

| **SES ->Pathways** | **Pooled data** | | **Urban** | | **Rural** | |
| --- | --- | --- | --- | --- | --- | --- |
|  | Coefficients | P\|z\| | Coefficient | P\|z\| | Coefficients | P\|z\| |
| Mother education | 0.169 | 0.016 | 0.321 | 0.202 | -0.194 | 0.418 |
| Father education | -0.275 | 0.000 | -0.421 | 0.028 | -0.248 | 0.315 |
| Occupation | 0.287 | 0.005 | 0.279 | 0.321 | 0.189 | 0.004 |
| Log of household size | -0.118 | 0.264 | 0.253 | 0.389 | -0.253 | 0.174 |
| Log of household Income | -0.085 | 0.042 | -0.109 | 0.009 | -0.185 | 0.432 |
| **Clinic Visits** |  |  |  |  |  |  |
| Mother education | 0.029 | 0.643 | 0.020 | 0.832 | 0.022 | 0.791 |
| Father education | -0.013 | 0.838 | -0.098 | 0.283 | 0.047 | 0.589 |
| Occupation | 0.023 | 0.791 | 0.206 | 0.204 | 0.010 | 0.935 |
| Log of household size | 0.055 | 0.606 | -0.053 | 0.767 | 0.145 | 0.278 |
| Log of household Income | -0.079 | 0.022 | -0.088 | 0.149 | -0.084 | 0.047 |
| **Drinking Boiled water** |  |  |  |  |  |  |
| Mother education | -0.076 | 0.056 | -0.070 | 0.27 | -0.070 | 0.171 |
| Father education | -0.125 | 0.002 | -0.087 | 0.165 | -0.115 | 0.03 |
| Occupation | 0.131 | 0.018 | 0.228 | 0.038 | 0.032 | 0.664 |
| Log of household size | 0.112 | 0.100 | 0.185 | 0.127 | 0.071 | 0.381 |
| Log of household Income | -0.075 | 0.001 | -0.129 | 0.002 | -0.053 | 0.041 |
