# Supplementary material for: Rural–urban disparities in child nutrition in Tabora, Tanzania: a socioeconomic decomposition and implications for food security policy
Source: Front Nutr. 2026 Jul 20;13:1800873. doi: 10.3389/fnut.2026.1800873 (PMC13430998; doi:10.3389/fnut.2026.1800873)
Supplement: Supplementary file 3 [file Table_3.docx]

**Table A3: Decomposition technique results**

| **haz** | **Coef.** | | **Robust**  **Std. Err.** | **z** | | **P>z** | | **[95% Conf.** | | **Interval]** |
| --- | --- | --- | --- | --- | --- | --- | --- | --- | --- | --- |
| **group_1** | -1.8860 | | 0.0945 | -19.94 | | 0.000 | | -2.0714 | | -1.7006 |
| **group_2** | -1.2001 | | 0.1308 | -9.17 | | 0.000 | | -1.4565 | | -0.9436 |
| **difference** | -0.6859 | | 0.1614 | -4.25 | | 0.000 | | -1.0023 | | -0.3695 |
| **explained** | -0.4913 | | 0.1191 | -4.12 | | 0.000 | | -0.7248 | | -0.2578 |
| **unexplained** | -0.1945 | | 0.1159 | -1.68 | | 0.093 | | -0.4219 | | 0.0327 |
| **Explained** | | | | | | | | | | |
| Mothered1 | 0.0032 | | 0.0491 | 0.070 | | 0.948 | | -0.0931 | | 0.0995 |
| Mothered2 | 0.0178 | | 0.0617 | 0.290 | | 0.772 | | -0.1032 | | 0.1390 |
| Mothered3 | 0.0300 | | 0.0864 | 0.350 | | 0.728 | | -0.1392 | | 0.1790 |
| Fathered1 | -0.0786 | | 0.0397 | -1.980 | | 0.048 | | -0.1564 | | -0.0007 |
| Fathered2 | -0.0403 | | 0.0783 | -0.510 | | 0.607 | | -0.1939 | | 0.1132 |
| Fathered3 | 0.0578 | | 0.0708 | 0.820 | | 0.415 | | -0.0810 | | 0.1966 |
| occupation1 | 0.0324 | | 0.0921 | 0.350 | | 0.724 | | -0.1481 | | 0.2131 |
| Difficulty1 | 0.0030 | | 0.0087 | 0.350 | | 0.730 | | -0.0141 | | 0.0201 |
| Difficulty2 | -0.0069 | | 0.0149 | -0.460 | | 0.644 | | -0.0362 | | 0.0224 |
| Difficulty3 | -0.0235 | | 0.0278 | -0.850 | | 0.397 | | -0.0780 | | 0.0309 |
| Clinic1 | -0.0089 | | 0.0142 | -0.630 | | 0.530 | | -0.0368 | | 0.0189 |
| Clinic2 | 0.0061 | | 0.0133 | 0.460 | | 0.649 | | -0.0201 | | 0.0321 |
| Nutrition1 | -0.0692 | | 0.0442 | -1.560 | | 0.118 | | -0.1561 | | 0.0175 |
| Nutrition2 | -0.0992 | | 0.0478 | -2.070 | | 0.038 | | -0.1930 | | -0.0054 |
| Drinking1 | -0.1422 | | 0.0577 | -2.460 | | 0.014 | | -0.2554 | | -0.0291 |
| Ininc | -0.1814 | | 0.0783 | -2.320 | | 0.021 | | -0.3349 | | -0.0279 |
| Inhhsize | 0.0086 | | 0.0380 | 0.230 | | 0.821 | | -0.0659 | | 0.0832 |
| **Unexplained** | | | | | | | | | | |
| Mothered1 | -0.2645 | | 0.0955 | -2.770 | | 0.006 | | -0.4517 | | -0.0774 |
| Mothered2 | -0.6636 | | 0.3611 | -1.840 | | 0.066 | | -1.3714 | | 0.0441 |
| Mothered3 | -0.1948 | | 0.1221 | -1.600 | | 0.111 | | -0.4342 | | 0.0444 |
| Fathered1 | 0.0428 | | 0.0667 | 0.640 | | 0.520 | | -0.0878 | | 0.1736 |
| Fathered2 | 0.1189 | | 0.4455 | 0.270 | | 0.789 | | -0.7543 | | 0.9922 |
| Fathered3 | 0.0659 | | 0.1379 | 0.480 | | 0.632 | | -0.2043 | | 0.3363 |
| occupation1 | 0.0947 | | 0.2421 | 0.390 | | 0.696 | | -0.3798 | | 0.5693 |
| Difficulty1 | 0.1151 | | 0.0639 | 1.800 | | 0.072 | | -0.0102 | | 0.2405 |
| Difficulty2 | 0.0300 | | 0.0522 | 0.580 | | 0.565 | | -0.0722 | | 0.1323 |
| Difficulty3 | 0.1119 | | 0.1237 | 0.900 | | 0.366 | | -0.1305 | | 0.3544 |
| Clinic1 | -0.4068 | | 0.3765 | -1.080 | | 0.280 | | -1.1448 | | 0.3311 |
| Clinic2 | -0.0354 | | 0.0381 | -0.930 | | 0.352 | | -0.1102 | | 0.0392 |
| Nutrition1 | -0.3009 | | 0.1721 | -1.750 | | 0.080 | | -0.6384 | | 0.0365 |
| Nutrition2 | 0.0807 | | 0.0695 | 1.160 | | 0.246 | | -0.0555 | | 0.2169 |
| Drinking1 | 0.0299 | | 0.1374 | 0.220 | | 0.827 | | -0.2393 | | 0.2992 |
| Ininc | -1.2862 | | 1.5764 | -0.820 | | 0.415 | | -4.3761 | | 1.8034 |
| Inhhsize | 0.1956 | | 0.5879 | 0.330 | | 0.739 | | -0.9568 | | 1.3480 |
| _cons | 2.0720 | | 2.2188 | 0.930 | | 0.350 | | -2.2768 | | 6.4209 |
| Blinder-Oaxaca decomposition | | Number of obs | | | = | | 490 | |  | |
|  | | Model | | | = | | linear | |  |  |
| Group 1: residenc = 1 | | N of obs 1 | | | = | | 193 | |  |  |
| Group 2: residenc = 2 | | N of obs 2 | | | = | | 297 | |  |  |
