# Supplementary material for: Rural–urban disparities in child nutrition in Tabora, Tanzania: a socioeconomic decomposition and implications for food security policy
Source: Front Nutr. 2026 Jul 20;13:1800873. doi: 10.3389/fnut.2026.1800873 (PMC13430998; doi:10.3389/fnut.2026.1800873)
Supplement: Supplementary file 4 [file Table_4.docx]

**Model diagnostic for Instrumental variable regression**

**Table A4: Test for Endogeneity**

| **Tests** | **Test Scores** | **P - Value** |
| --- | --- | --- |
| Durbin (score) chi2(1) | 23.012*** | 0.0004 |
| Wu-Hausman | 7.022*** | 0.0023 |

**** p<0.01, ** p<0.05, * p<0.1*
