# Supplementary material for: Rural–urban disparities in child nutrition in Tabora, Tanzania: a socioeconomic decomposition and implications for food security policy
Source: Front Nutr. 2026 Jul 20;13:1800873. doi: 10.3389/fnut.2026.1800873 (PMC13430998; doi:10.3389/fnut.2026.1800873)
Supplement: Supplementary file 5 [file Table_5.docx]

**Model diagnostic for Instrumental variable regression**

**Table A5: Summary statistics for First Stage regression**

| **Variable** | **R-square** | **Adjusted**  **R-square** | **Partial**  **R-square** | **Prob > F** |
| --- | --- | --- | --- | --- |
| Distance nearest health facility | 0.7427 | 0.7373 | 0.7099 | 0.0003 |
