# Supplementary material for: Rural–urban disparities in child nutrition in Tabora, Tanzania: a socioeconomic decomposition and implications for food security policy
Source: Front Nutr. 2026 Jul 20;13:1800873. doi: 10.3389/fnut.2026.1800873 (PMC13430998; doi:10.3389/fnut.2026.1800873)
Supplement: Supplementary file 6 [file Table_6.docx]

**Model diagnostic for Instrumental variable regression**

**Table A6: Instrument Strength by Eigenvalue Statistic**

|  | **Critical Values** | | | |
| --- | --- | --- | --- | --- |
| 2SLS relative bias | 5% | 10% | 20% | 30% |
|  | 45.34 | 43.01 | 40.09 | 35.99 |
|  | 10% | 15% | 20% | 25% |
| 2SLS Size of nominal 0.05 Wald test | 40.67 | 37.15 | 35.26 | 31.12 |
| LIML Size of nominal 5% Wald test | 33.92 | 26.04 | 24.08 | 20.02 |

**eigenvalue statistic = 72.03**
